# Ethnic inequities in use of breast conserving surgery and radiation therapy in Aotearoa/New Zealand: which factors contribute?

**DOI:** 10.1007/s10549-024-07289-8

**Published:** 2024-03-27

**Authors:** Leah Boyle, Ross Lawrenson, Vili Nosa, Ian Campbell, Sandar Tin Tin

**Affiliations:** 1 Cancer Epidemiology Unit, Oxford Population Health, The University of Oxford, Oxford, UK; 2 University of Waikato, Hamilton, New Zealand; 3 Waikato District Health Board, Hamilton, New Zealand; 4 Faculty of Medical and Health Sciences, University of Auckland, Auckland, New Zealand; 5 Department of Surgery, Faculty of Health Sciences, University of Auckland, Auckland, New Zealand; 6 Epidemiology and Biostatistics, School of Population Health, University of Auckland, Auckland, New Zealand

**Keywords:** Breast cancer, Surgery, Mastectomy, Breast-conserving surgery, Radiotherapy, Inequities

## Abstract

**Purpose:**

Aotearoa/New Zealand (NZ) faces ethnic inequities with respect to breast cancer survival and treatment. This study establishes if there are ethnic differences in (i) type of surgery and (ii) receipt of radiotherapy (RT) following breast conserving surgery (BCS), among women with early-stage breast cancer in NZ.

**Methods:**

This analysis used *Te Rēhita Mate Ūtaetae* (Breast Cancer Foundation National Register), a prospectively maintained database of breast cancers from 2000 to 2020. Logistic regression models evaluated ethnic differences in type of surgery (mastectomy or BCS) and receipt of RT with sequential adjustment for potential contributing factors. Subgroup analyses by treatment facility type were undertaken.

**Results:**

Of the 16,228 women included, 74% were NZ European (NZE), 10.3% were Māori, 9.4% were Asian and 6.2% were Pacific. Over one-third of women with BCS-eligible tumours received mastectomy. Asian women were more likely to receive mastectomy than NZE (OR 1.62; 95% CI 1.39, 1.90) as were wāhine Māori in the public system (OR 1.21; 95% CI 1.02, 1.44) but not in the private system (OR 0.78; 95% CI 0.51, 1.21). In women undergoing BCS, compared to NZE, Pacific women overall and wāhine Māori in the private system were, respectively, 36 and 38% less likely to receive RT (respective OR 0.64; 95% CI 0.50, 0.83 and 0.62; 95% CI 0.39, 0.98).

**Conclusion:**

A significant proportion of women with early-stage breast cancer underwent mastectomy and significant ethnic inequities exist. Modern guidelines encourage BCS + RT. In NZ, this outcome must be carefully monitored by ethnicity to facilitate equitable surgical management of early-stage breast cancer.

**Supplementary Information:**

The online version contains supplementary material available at 10.1007/s10549-024-07289-8.

## Introduction

Breast cancer is the most common cancer for women globally, with over two million new cases diagnosed in 2020 [1]. Aotearoa/New Zealand (NZ) has one of the highest incidences globally, with an age-standardized rate of 93.0 per 100,000 population [2]. This corresponds to one breast cancer diagnosis among every nine NZ women [3]. This high incidence is compounded by significant ethnic inequities in breast cancer survival, disproportionately affecting Māori (Indigenous people) and Pacific (migrants from Pacific Islands or descendants of migrants from Pacific islands) people, who comprise 16 and 8% of the NZ population, respectively [4–6].

Causes of this ethnic inequity in breast cancer survival are complex, encompassing a range of demographic (e.g. socioeconomic status), tumour (e.g. stage and histological type) and health system (e.g. access to public or private care) factors [4, 7, 8]. These health inequities in NZ have been shaped by the impact of colonization [9]. Acknowledging this aspect of NZ’s history is crucial to understanding, addressing and eliminating these disparities. For breast cancer, key contributors to the survival disadvantage experienced by wāhine Māori and Pacific women (compared to NZE women), namely deprivation and late stage at diagnosis, ultimately reflect the downstream consequences of colonization [4, 9].

Examining this ethnic inequity also requires consideration of the treatment for breast cancer - surgery is the primary treatment for most early-stage (1–3a) breast cancers. The New Zealand Management of Early Breast Cancer guidelines, introduced in 2009, recommend offering women the choice of breast conserving surgery (e.g. wide local excision (WLE) or lumpectomy) with radiotherapy (BCS + RT) or mastectomy with or without reconstruction for early-stage breast cancers, if the tumour is unilateral, unifocal and clinically the person has adequate breast volume to facilitate acceptable cosmesis [10]. Key randomized controlled trials demonstrated that BCS + RT has equivalent disease-free survival, and overall survival to mastectomy for women with early-stage breast cancer and therefore many early guidelines recommended that women are offered the choice of surgery type [11, 12]. Furthermore, two recent meta-analyses-one including over 1.5 million women-report a survival benefit from BCS + RT, compared to mastectomy [13, 14]. Additionally, BCS + RT is less invasive and does not require breast reconstruction; it has been associated with a shorter hospital stay, fewer post-operative complications and higher patient satisfaction [10, 13, 15, 16]. BCS + RT therefore represents the favourable option for women with early-stage breast cancer, if it is technically feasible to perform [14].

Despite the earlier opinion of the oncological equivalence of BCS + RT, prior research demonstrated ethnic inequities with respect to surgery type. In NZ, in the Waikato and Auckland regions, wāhine Māori diagnosed with breast cancer between 1992 and 2012, had higher mastectomy rates compared to NZ European (NZE) women, even when BCS eligible [17, 18]. BCS + RT is also shown to be less likely among Asian women in the Auckland region [17]. However these earlier studies focus on two regions of NZ only and it is unclear if these findings still persist or exist in other regions of NZ. Additionally, the oncological equivalence of BCS to mastectomy is dependent upon receipt of adjuvant RT to achieve local disease control–however, there are limited studies examining ethic differences in the receipt of RT following BCS, with no studies including women diagnosed with breast cancer since 2015 [19].

This paper will use *Te Rēhita Mate Ūtatatae* (Breast Cancer Foundation National Register) to establish whether there are (i) ethnic differences in women with early-stage (1–3a) breast cancer, from 2000 to 2020 in type of surgery (BCS vs. mastectomy), (ii) ethnic differences in receipt of RT in women undergoing BCS and finally examine the impact of demographic, tumour and health pathway/system factors on these potential associations.

## Methods

### Study design and data sources

This retrospective study used the data from *Te Rēhita Mate Ūtaetae* (Breast Cancer Foundation National Register), a prospectively maintained database which records all primary breast cancer diagnoses in four large tertiary centres in NZ—Auckland, Waikato, Christchurch and Wellington. This comprises two-thirds of the country’s population and is representative of 63% of national breast cancer cases [20]. *Te Rēhita Mate Ūtaetae* is shown to be more comprehensive than national databases with detailed information on patient demographics, date and mode of diagnosis, tumour characteristics and treatment factors [20, 21].

### Study population

**Fig. 1:**
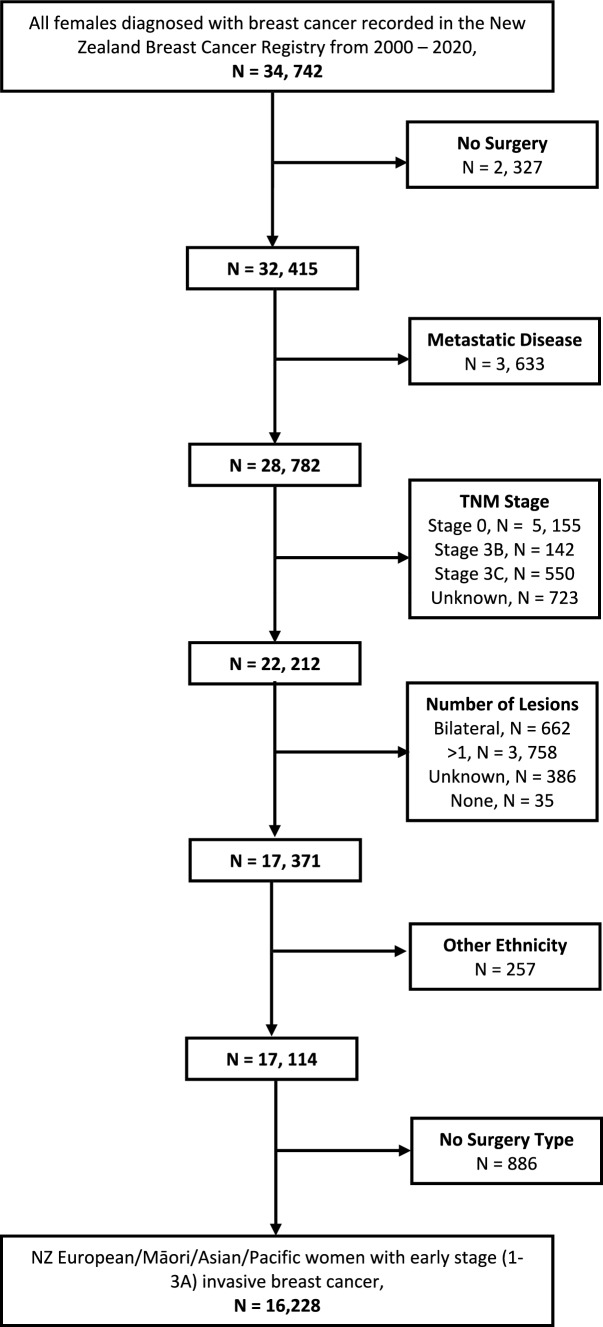
Sample restriction flowchart

This study included the 16,228 women who were diagnosed with histologically confirmed early-stage (1–3a) primary invasive breast cancer between 1 June 2000 and 31 December 2020 and received surgery as their primary cancer treatment. Women with bilateral, multi-focal, stage 3b and 3c or metastases were excluded as these women are not BCS eligible, or do not have surgery as a primary treatment (Fig. 1) [10]. 886 women with no type of surgery recorded and 2327 women who did not receive surgery were also excluded.

### Variables of interest

The exposure of interest was ethnicity categorized as NZE, Māori, Asian or Pacific. Ethnicity in the register is sourced from the MOH through linkage with individual National Health Identifier numbers. The register allows for up to three ethnicities to be selected [20]. As per the NZ MOH ‘HISO 10001:2017 Ethnicity Data Protocol’, patients with more than one recorded ethnicity were allocated to a single ethnic group in order of priority: Māori, Pacific, Asian and European/other [22]. ‘Other’ ethnicity comprises 1.5% of patients in the register and was excluded from this analysis, in alignment with prior register analyses of ethnicity-based outcomes [20].

The primary outcome was type of surgery: BCS (which included lumpectomy or wide local excision) versus mastectomy. The secondary outcome was receipt of RT. For this outcome, the sample was restricted to the 10,384 women receiving BCS.

**Fig. 2:**
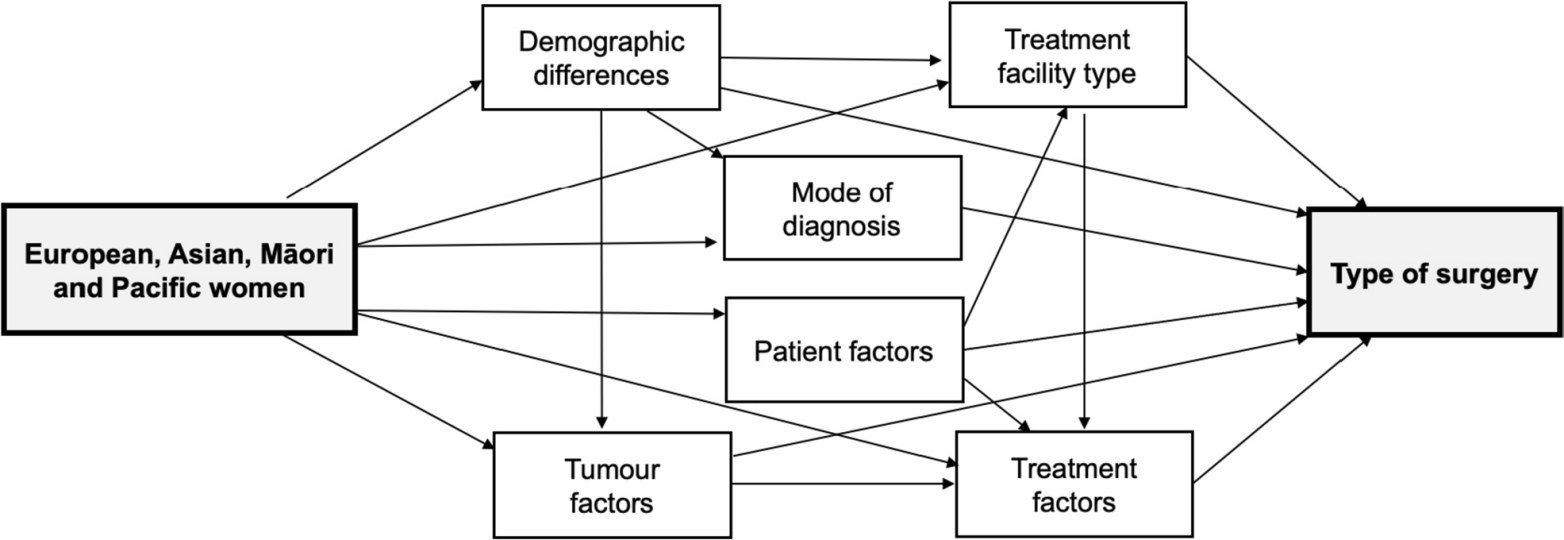
Conceptual framework displaying potential contributing factors on the ethnicity-type of surgery associations, for women with breast cancer in New Zealand

Other variables for analysis which may contribute to the association between ethnicity and type of surgery were selected a priori based on prior literature (Fig. 2) [4, 7, 17, 18, 20]. Those included in the models were: (1) demographic factors—age (< 45 years, ≥ 45 to ≤ 69 years (screening age in NZ), > 69 years), region, area of residence (rural/urban), NZ deprivation index (1, least deprived to 10, most deprived, in quintiles), (2) mode of diagnosis (screened/symptomatic which includes public and private), (3) tumour biology factors—TNM stage (1–3a)**,** grade (low, intermediate, high, unknown), histology (ductal, lobular, mixed, other, unknown), oestrogen and progesterone receptors (ER and PR) and human epidermal growth factor receptors (HER), (4) treatment facility (public/private) and (5) treatment factors—radiotherapy and systemic therapy (see Supplementary Table S1 for detail on variable categorization).

### Statistical analyses

Descriptive analyses summarized the data by ethnicity; the data was presented as proportions (%) and differences across four ethnic groups were assessed using chi-squared (*χ*^2^) tests.

Using NZE as the reference group, multivariate logistic regression models were built to obtain odds ratio (OR) with 95% confidence intervals (CI) for (i) ethnicity and type of surgery (BCS/mastectomy) and (ii) ethnicity and receipt of RT. The models were adjusted in a step wise fashion, in five domains to build a total of five models for each outcome. Model one included adjustment for demographic factors, model two additionally included adjustment for mode of diagnosis, model three included adjustment for tumour factors, model four included adjustment for treatment facility and then the maximally adjusted model, model five, included adjustment for treatment factors (excluding the RT covariate for outcome two).

Subgroup analyses by mode of diagnosis and by treatment facility type were undertaken for both outcomes. Those with ‘unknown’ recorded for the latter were excluded from this subgroup analysis (*n* = 252); there was no missing data for mode of diagnosis. *χ*^2^ for heterogeneity were obtained to determine whether the risk estimates from these subgroup analyses were different. Sensitivity analysis was undertaken with comorbidity added to maximally adjusted models for type of surgery and receipt of RT, using the Charlson Comorbidity Index (CCI) by restricting the sample to the women with CCI recorded (4084 and 2543, respectively). This is a validated index which measures the presence of up to 19 comorbidities and weights them according to their associated mortality risk to obtain a score, categorized as: 0, 1–2, 3–4 and ≥ 5 [23]. A *p*-value of 0.05 was considered statistically significant. Data was analysed in Stata MP version 17.0 and SAS version 9.4.

## Results

### Participant characteristics

Of the 16,288 women included in this analysis, 74.1% were NZE, 9.4% were Asian, 10.2% were Māori and 6.2% were Pacific (Table 1). Most were in the screening age group (≥ 45 to ≤ 69 years). Asian and Pacific ethnicities had the greatest proportion of younger women < 45 years with breast cancer (20.6 and 17.3%, respectively). Wāhine Māori and Pacific women were more likely to live in deprived areas, with 34.3% and 45.7% living in decile 9–10 areas, respectively (compared to 8.80% of NZE). They were also more likely to have a later stage at diagnosis with the greatest proportion of stage 2b cancers (14.2 and 16.2%, respectively, compared to 10.6% among NZE) and 3a cancers (8.1 and 10.7% respectively, compared to 5.7% among NZE) and were markedly less likely to receive treatment in a private facility (12.4 and 9.2%, respectively), compared to NZE (40.1%) and Asian (31.5%) women.

**Table 1:** Baseline demographic, tumour and treatment characteristics by ethnicity

Characteristic (%)	Total (*n* = 16, 228)	NZ European (*n* = 12, 019)	Māori (*n* = 1, 678)	Asian (*n* = 1,521)	Pacific (*n* = 1, 010)
Age (years)					
< 45	11.6	9.7	13.6	20.6	17.3
≥ 45 to ≤ 69	69.6	68.0	77.7	70.2	73.5
> 69	18.8	22.3	8.8	9.1	9.2
Region					
Auckland	59.2	55.4	51.7	81.7	82.8
Waikato	17.6	18.7	29.9	4.7	4.9
Christchurch	11.2	13.4	6.0	5.3	2.3
Wellington	12.0	12.6	12.5	8.3	10.1
NZ deprivation index					
1–2 (least deprived)	22.0	25.1	9.1	22.0	6.4
3–4	20.9	22.7	11.7	25.4	8.4
5–6	20.5	21.8	15.5	21.0	11.8
7–8	18.8	17.7	26.3	16.6	23.1
9–10 (most deprived)	14.2	8.8	34.3	13.7	45.7
Unknown	3.7	4.0	3.1	1.3	4.6
Area of residence					
Urban	86.5	84.8	84.4	96.4	94.4
Rural	19.9	11.2	12.5	2.3	1.1
Unknown	3.6	3.9	3.1	1.3	4.6
Mode of diagnosis					
Screen-detected	47.7	48.2	50.1	42.3	47.0
Symptomatic	52.3	51.9	49.9	57.7	53.0
TNM stage					
1a	52.4	54.4	48.2	50.3	39.4
1b	2.9	3.0	3.1	2.5	2.4
2a	26.3	25.7	26.0	28.2	30.6
2b	11.9	11.0	14.2	12.4	16.6
3a	6.6	5.9	8.3	6.6	11.0
Cancer grade					
Low	25.6	26.4	24.3	23.7	19.9
Intermediate	46.6	46.6	50.4	43.5	45.7
High	26.8	26.0	24.0	31.5	33.2
Unknown	1.0	0.9	1.4	1.3	1.2
Histology					
Ductal	80.2	79.4	81.8	84.2	81.6
Lobular	10.1	11.0	9.2	5.7	7.0
Mixed	2.6	2.5	3.0	3.1	3.1
Other	6.2	6.3	4.8	6.1	6.9
Unknown	0.9	0.9	1.1	0.9	1.4
Receptors					
ER+ /PR+	55.2	55.0	52.0	56.2	61.6
ER+ /PR−	9.0	9.5	6.1	10.7	55.4
ER−/PR+	0.8	0.8	0.7	1.3	0.5
ER−/PR−	12.2	12.2	9.9	13.0	14.6
Unknown	22.7	22.4	31.4	19.0	18.0
HER					
Negative	75.4	75.4	76.5	77.6	70.4
Equivocal	0.2	0.3	0.1	0.2	0.2
Positive	12.3	11.2	14.2	14.5	19.1
Unknown	12.0	13.1	9.2	7.8	10.3
Treatment facility					
Public	63.4	57.6	86.9	65.6	90.3
Private	35.0	40.7	12.5	32.3	9.3
Unknown	1.6	1.7	0.7	2.0	0.4
Locoregional treatment					
BCS no RT	8.6	8.8	8.3	6.7	9.4
BCS with RT	54.7	56.7	54.2	45.7	45.6
Mastectomy no RT	25.6	24.5	23.2	34.4	66.7
Mastectomy with RT	11.0	9.9	14.2	13.2	15.0
Systemic therapy					
Yes	73.2	71.7	78.7	76.1	76.7
No	26.8	28.3	21.3	23.9	23.3

Ethnic differences for all characteristics statistically significant with *p* < 0.001 in *χ*^2^ tests

*TNM* tumour node metastasis stage, *ER* oestrogen receptor, *PR* progesterone receptor, *HER* human epidermal growth factor receptor, *BCS* breast conserving surgery, *RT* radiotherapy

### Type of surgery

Sixty-three-point four percent of women received BCS. In the unadjusted model, compared to NZE women, all other ethnicities were more likely to receive mastectomy (OR 1.14; 95% CI 1.02, 1.27 for wāhine Māori, OR 1.73; 95% CI 1.55, 1.93 for Asian women, and OR 1.56; 95% CI 1.37, 1.77 for Pacific women) (Table 2). For wāhine Māori, the OR increased to 1.34 (95% CI 1.19, 1.50) after the addition of demographic factors and mode of diagnosis and then decreased to 1.16 (95% CI 1.02, 1.31) after the addition of tumour factors and treatment facility type. In the maximally adjusted model, there remained no statistically significant difference in odds of mastectomy for wāhine Māori (OR 1.15; 95% CI 0.98, 1.34) or Pacific women (OR 0.96; 95% CI 0.79, 1.17). For Pacific women, tumour factors, treatment facility type and treatment factors contributed most to the reduction in risk difference (109.7% attenuation; 95% CI 82.9%, 151.4%). Asian women were the only ethnicity more likely to receive mastectomy than NZE in the maximally adjusted model (OR 1.62; 95% CI 1.39, 1.90) and factors included did not contribute significantly to a reduction in OR. Supplementary Table S2 displays the associations for all the covariates included in the maximally adjusted models.

**Table 2:** Multivariate logistic regression models for odds of mastectomy versus breast conserving surgery by ethnicity

Model	Additional variables in model^a^	NZ European (*n* = 12,019)	Māori (*n* = 1678)	Asian (*n* = 1521)	Pacific (*n* = 1010)
		Reference	OR (95% CI)		
Unadjusted		1.00	1.14 (1.02, 1.27)	1.73 (1.55, 1.93)	1.56 (1.37, 1.77)
1. Unadjusted + demographics	Age	1.00	1.29 (1.16, 1.44)	1.89 (1.69, 2.11)	1.73 (1.51, 1.97)
	Region	1.00	1.43 (1.28, 1.59)	1.83 (1.64, 2.05)	1.71 (1.50, 1.96)
	NZ Dep Index	1.00	1.35 (1.20, 1.51)	1.84 (1.65, 2.06)	1.58 (1.37, 1.82)
	Area of residence	1.00	1.34 (1.20, 1.51)	1.85 (1.65, 2.07)	1.58 (1.37, 1.82)
2. Model 1 + mode of diagnosis	Mode of diagnosis	1.00	1.34 (1.19, 1.50)	1.77 (1.57, 1.98)	1.55 (1.35, 1.80)
3. Model 2 + tumour factors	Stage	1.00	1.23 (1.09, 1.39)	1.81 (1.60, 2.04)	1.31 (1.13, 1.53)
	Grade	1.00	1.23 (1.09, 1.39)	1.80 (1.59, 2.03)	1.30 (1.11, 1.51)
	Histology	1.00	1.24 (1.10, 1.40)	1.84 (1.63, 2.08)	1.33 (1.14, 1.55)
	ER/PR	1.00	1.25 (1.10, 1.41)	1.87 (1.66, 2.11)	1.35 (1.16, 1.57)
	HER	1.00	1.25 (1.10, 1.41)	1.88 (1.67, 2.13)	1.32 (1.13, 1.54)
4. Model 3 + treatment facility	Treatment facility	1.00	1.16 (1.02, 1.31)	1.80 (1.59, 2.04)	1.21 (1.03, 1.41)
5. Model 4 + treatment factors	Radiotherapy	1.00	1.14 (0.97, 1.33)	1.64 (1.40,1.92)	0.96 (0.79, 1.17)
	Systemic	1.00	1.15 (0.98, 1.34)	1.62 (1.39, 1.90)	0.96 (0.79, 1.17)

*OR* odds ratio, *CI* confidence interval, *NZ Dep Index* New Zealand deprivation index, *ER* oestrogen receptor, *PR* progesterone receptor, *HER* human epidermal growth factor receptor

^a^Variables are categorized as follows: age; < 45 years, ≥ 45 to ≤ 69 years (women eligible for BSA) and > 69 years, region; Auckland, Waikato, Christchurch, Wellington, NZ Dep Index; decile 1—least deprived to decile 10—most deprived, area of residence; rural or urban, mode of diagnosis; screened or symptomatic, stage; using AJCC 7th edition TNM staging, grade; 1—low to 3—high, histology; ductal, lobular, mixed, other, ER/PR; ER+/PR+, ER+/PR−, ER−/PR+, ER−/PR−, unknown, HER; negative, equivocal, positive, unknown, treatment facility; public or private, radiotherapy; radiotherapy or no radiotherapy, systemic; systemic treatment(chemotherapy, hormone therapy or biologics) or no systemic treatment

### Receipt of radiotherapy in women undergoing breast conserving surgery

Of the 10,284 women receiving BCS, 86.4% received RT. In maximally adjusted models, Pacific women were 36% less likely to receive RT than NZE with an OR of 0.64; 95% CI 0.50, 0.83 (Table 3). For other ethnicities compared to NZE, there were no significant differences in receipt of RT–Māori OR 0.85 (95% CI 0.69, 1.04) and Asian OR 0.95 (95% CI 0.75, 1.19).

**Table 3:** Multivariate logistic regression models for receipt of radiotherapy in women receiving breast conserving surgery by ethnicity

Model	Additional variables in model^a^	NZ European (*n* = 7881)	Māori (*n* = 1050)	Asian (*n* = 797)	Pacific (*n* = 556)
		Reference	OR (95% CI)		
Unadjusted		1.00	1.01 (0.84, 1.22)	1.06 (0.85, 1.32)	0.75 (0.60, 0.95)
1. Unadjusted + demographics	Age	1.00	0.89 (0.73, 1.07)	0.92 (0.74, 1.15)	0.65 (0.52, 0.83)
	Region	1.00	0.89 (0.74, 1.09)	0.98 (0.78, 1.22)	0.70 (0.55, 0.88)
	NZ Dep Index	1.00	0.88 (0.72, 1.07)	0.97 (0.78, 1.22)	0.69 (0.54, 0.88)
	Area of residence	1.00	0.88 (0.72, 1.07)	0.98 (0.78, 1.22)	0.69 (0.54, 0.88)
2. Model 1 + mode of diagnosis	Mode of diagnosis	1.00	0.88 (0.72, 1.07)	0.98 (0.79, 1.23)	0.69 (0.54, 0.88)
3. Model 2 + tumour factors	Stage	1.00	0.86 (0.71, 1.06)	0.99 (0.79, 1.23)	0.66 (0.51, 0.84)
	Grade	1.00	0.87 (0.71, 1.06)	0.98 (0.78, 1.23)	0.65 (0.51, 0.83)
	Histology	1.00	0.87 (0.71, 1.06)	0.98 (0.78, 1.23)	0.65 (0.51, 0.83)
	ER/PR	1.00	0.87 (0.71, 1.06)	0.99 (0.79, 1.24)	0.65 (0.51, 0.84)
	HER	1.00	0.87 (0.71, 1.07)	0.99 (0.79, 1.24)	0.65 (0.51, 0.84)
4. Model 3 + treatment facility	Treatment facility	1.00	0.87 (0.71, 1.06)	0.99 (0.79, 1.24)	0.65 (0.51, 0.83)
5. Model 4 + treatment factors	Systemic	1.00	0.85 (0.69, 1.04)	0.95 (0.75, 1.19)	0.64 (0.50, 0.83)

*OR* odds ratio, *CI* confidence interval, *NZ Dep Index* New Zealand deprivation index, *ER* oestrogen receptor, *PR* progesterone receptor, *HER* human epidermal growth factor receptor

^a^Variables are categorized as follows: age; < 45 years, ≥ 45 to ≤ 69 years (women eligible for BSA) and > 69 years, region; Auckland, Waikato, Christchurch, Wellington, NZ Dep Index; decile 1—least deprived to decile 10—most deprived, area of residence; rural or urban, mode of diagnosis; screened or symptomatic, stage; using AJCC 7th edition TNM staging, grade; 1—low to 3—high, histology; ductal, lobular, mixed, other, ER/PR; ER+/PR+, ER+/PR−, ER−/PR+, ER−/PR−, unknown, HER; negative, equivocal, positive, unknown, systemic; systemic treatment(chemotherapy, hormone therapy or biologics) or no systemic treatment

### Subgroup analysis by mode of diagnosis

Forty-seven-point seven percent of women were diagnosed through screening. There were no significant differences in odds of mastectomy by mode of diagnosis for any ethnicity; greater deprivation, stage and public hospital treatment were all key contributing factors for more mastectomies in both groups (Table 4).

**Table 4:** Subgroup analysis for odds of mastectomy versus breast conserving surgery by ethnicity for screened and symptomatic mode of diagnosis

Model	Additional variables in model^a^	Screened (*n* = 7747) OR (95% CI)				Symptomatic (*n* = 8481) OR (95% CI)			
		NZ European (*n* = 5787)	Māori (*n* = 841)	Asian (*n* = 644)	Pacific (*n* = 475)	NZ European (*n* = 6232)	Māori (*n* = 837)	Asian (*n* = 877)	Pacific (*n* = 535)
Unadjusted		1.00	1.19 (1.00, 1.41)	1.96 (1.64, 2.33)	1.67 (1.36, 2.04)	1.00	1.17 (1.01, 1.35)	1.53 (1.32, 1.76)	1.54 (1.29, 1.84)
1. Unadjusted + demographics	Age	1.00	1.23 (1.03, 1.45)	2.00 (1.68, 2.39)	1.72 (1.40, 2.11)	1.00	1.31 (1.13, 1.52)	1.72 (1.43, 2.06)	1.71 (1.43, 2.06)
	Region	1.00	1.36 (1.14, 1.62)	1.88 (1.57, 2.25)	1.65 (1.34, 2.04)	1.00	1.46 (1.26, 1.69)	1.66 (1.43, 1.92)	1.69 (1.41, 2.04)
	NZ Dep Index	1.00	1.27 (1.06, 1.52)	1.90 (1.58, 2.27)	1.51 (1.21, 1.88)	1.00	1.38 (1.19, 1.62)	1.66 (1.43, 1.93)	1.57 (1.30, 1.90)
	Area of residence	1.00	1.26 (1.05, 1.51)	1.92 (1.60, 2.29)	1.52 (1.22, 1.89)	1.00	1.38 (1.19, 1.61)	1.66 (1.43, 1.93)	1.57 (1.30, 1.90)
2. Model 1 + tumour factors	Stage	1.00	1.17 (0.97 1.41)	1.92 (1.59, 2.31)	1.32 (1.05, 1.66)	1.00	1.27 (1.09, 1.49)	1.73 (1.47, 2.02)	1.29 (1.05, 1.58)
	Grade	1.00	1.16 (0.96, 1.40)	1.90 (1.57, 2.29)	1.29 (1.02, 1.62)	1.00	1.27 (1.08, 1.49)	1.72 (1.47, 2.02)	1.28 (1.05, 1.57)
	Histology	1.00	1.16 (0.96, 1.41)	1.95 (1.62, 2.36)	1.31 (1.04, 1.66)	1.00	1.29 (1.09, 1.51)	1.77 (1.51, 2.07)	1.32 (1.08, 1.61)
	ER/PR	1.00	1.18 (0.97, 1.42)	1.97 (1.63, 2.38)	1.34 (1.06, 1.69)	1.00	1.29 (1.09, 1.52)	1.80 (1.54, 2.11)	1.34 (1.10, 1.65)
	HER	1.00	1.18 (0.98, 1.43)	1.98 (1.64, 2.40)	1.34 (1.06, 1.70)	1.00	1.28 (1.09, 1.51)	1.82 (1.55, 2.13)	1.30 (1.06, 1.59)
3. Model 2 + treatment facility	Treatment facility	1.00	1.11 (0.92, 1.35)	1.91 (1.58, 2.31)	1.23 (0.97, 1.56)	1.00	1.18 (1.00, 1.40)	1.74 (1.48, 2.04)	1.19 (0.97, 1.46)
4. Model 3 + treatment factors	Radiotherapy	1.00	1.04 (0.80, 1.35)	1.80 (1.36, 2.36)	0.84 (0.61, 1.17)	1.00	1.19 (0.97, 1.45)	1.58 (1.31, 1.92)	1.00 (0.78, 1.30)
	Systemic	1.00	0.84 (0.61, 1.17)	1.79 (1.36, 2.36)	0.84 (0.61, 1.17)	1.00	1.20 (0.98, 1.46)	1.56 (1.29, 1.90)	1.00 (0.78, 1.30)

*OR* odds ratio, *CI* confidence interval, *NZ Dep Index* New Zealand deprivation index, *ER* oestrogen receptor, *PR* progesterone receptor, *HER* human epidermal growth factor receptor

^a^Variables are categorized as follows: age; < 45 years, ≥ 45 to ≤ 69 years (women eligible for BSA) and > 69 years, region; Auckland, Waikato, Christchurch, Wellington, NZ Dep Index; decile 1—least deprived to decile 10—most deprived, stage; using AJCC 7th edition TNM staging, grade; 1—low to 3—high, histology; ductal, lobular, mixed, other, ER/PR; ER+/PR+, ER+/PR−, ER−/PR+, ER−/PR−, unknown, HER; negative, equivocal, positive, unknown, treatment facility; public or private, radiotherapy; radiotherapy or no radiotherapy, systemic; systemic treatment(chemotherapy, hormone therapy or biologics) or no systemic treatment

In terms of receipt of RT in those undergoing BCS, screened Pacific women were 48% less likely to receive RT (OR 0.52; 95% CI 0.38, 0.73) compared to screened NZE women. There were no significant differences for other ethnicities. Age and tumour factors contributed most to this reduction in risk differential, with a reduction in OR from 0.65 (95% CI 0.48, 0.87) to 0.59 (95% CI 0.43, 0.79) and from 0.62 (95% CI 0.45, 0.86) to 0.57 (95% CI 0.41, 0.79) when these factors were added to the models, respectively. (Supplementary Table S3). *χ*^2^ for heterogeneity were non-significant for this subgroup analysis.

### Subgroup analysis by treatment facility type

Almost two-thirds (63.9%) of women received surgery in the public system. Compared to NZE, wāhine Māori were more likely to receive mastectomy in the public system but not in the private system, although the difference was not statistically significant. The odds of mastectomy did not differ by treatment facility type for Pacific and Asian women; Asian women were more likely to have mastectomy, compared to NZE, in both public and private systems (OR 1.65; 95% CI 1.35, 2.01 and OR 1.69; 95% CI 1.29, 2.21, respectively) (Table 5).

**Table 5:** Subgroup analysis for odds of mastectomy versus breast conserving by ethnicity in public and private treatment facilities

Model	Additional variables in model^a^	Public care (*n* = 10,290) OR (95% CI)				Private care (*n* = 5686) OR (95% CI)			
		NZ European (*n* = 6921)	Māori (*n* = 1458)	Asian (*n* = 999)	Pacific *(n* = 912)	NZ European (*n* = 4892)	Māori (*n* = 209)	Asian (*n* = 491)	Pacific (*n* = 94)
Unadjusted		1.00	1.03 (0.92, 1.16)	1.57 (1.37, 1.79)	1.32 (1.15, 1.52)	1.00	0.83 (0.61, 1.14)	1.99 (1.65, 2.40)	1.98 (1.31, 2.98)
1. Unadjusted + demographics	Age	1.00	1.24 (1.10, 1.39)	1.78 (1.55, 2.04)	1.57 (1.36, 1.81)	1.00	0.85 (0.61, 1.16)	2.02 (1.67, 2.45)	1.82 (1.20, 2.77)
	Region	1.00	1.36 (1.20, 1.54)	1.66 (1.44, 1.91)	1.48 (1.27, 1.71)	1.00	0.90 (0.64, 1.23)	2.00 (1.65, 2.42)	1.90 (1.25, 2.90)
	NZ Dep Index	1.00	1.30 (1.14, 1.47)	1.69 (1.47, 1.94)	1.38 (1.19, 1.61)	1.00	0.90 (0.65, 1.24)	2.02 (1.66, 2.45)	1.95 (1.27, 2.98)
	Area of residence	1.00	1.30 (1.14, 1.47)	1.70 (1.47, 1.95)	1.39 (1.19, 1.62)	1.00	0.90 (0.65, 1.25)	2.02 (1.67, 2.46)	1.95 (1.28, 2.98)
2. Model 1 + mode of diagnosis	Mode of diagnosis	1.00	1.27 (1.11, 1.45)	1.62 (1.40, 1.88)	1.35 (1.15, 1.59)	1.00	0.90 (0.65, 1.25)	1.92 (1.58, 2.34)	1.87 (1.21, 2.87)
3. Model 2 + tumour factors	Stage	1.00	1.17 (1.02, 1.34)	1.65 (1.42, 1.92)	1.14 (0.97, 1.36)	1.00	0.85 (0.60, 1.19)	2.02 (1.64, 2.48)	1.63 (1.03, 2.58)
	Grade	1.00	1.18 (1.02, 1.35)	1.64 (1.41, 1.92)	1.13 (0.96, 1.34)	1.00	0.82 (0.58, 1.17)	2.00 (1.63, 2.46)	1.63 (1.02, 2.57)
	Histology	1.00	1.18 (1.03, 1.36)	1.69 (1.45, 1.96)	1.15 (0.97, 1.36)	1.00	0.83 (0.59, 1.18)	2.05 (1.66, 2.52)	1.65 (1.05, 2.61)
	ER/PR	1.00	1.19 (1.04, 1.37)	1.71 (1.47, 2.00)	1.18 (1.00, 1.40)	1.00	0.84 (0.60, 1.20)	2.04 (1.66, 2.52)	1.64 (1.04, 2.60)
	HER	1.00	1.19 (1.04, 1.36)	1.72 (1.48, 2.00)	1.15 (0.97, 1.36)	1.00	0.84 (0.60, 1.20)	2.06 (1.67, 2.54)	1.63 (1.03, 2.60)
5. Model 3 + treatment factors	Radiotherapy	1.00	1.20 (1.01, 1.42)	1.67 (1.37, 2.03)	0.94 (0.76, 1.17)	1.00	0.78 (0.51, 1.20)	1.70 (1.29, 2.22)	1.12 (0.62, 2.00)
	Systemic	1.00	1.21 (1.02, 1.44)	1.65 (1.35, 2.01)	0.94 (0.76, 1.16)	1.00	0.78 (0.51, 1.21)	1.69 (1.29, 2.21)	1.12 (0.63, 2.01)

*OR* odds ratio, *CI* confidence interval, *NZ Dep Index* New Zealand deprivation index, *ER* oestrogen receptor, *PR* progesterone receptor, *HER* human epidermal growth factor receptor

^a^Variables are categorized as follows: age; < 45 years, ≥ 45 to ≤ 69 years (women eligible for BSA) and > 69 years, region; Auckland, Waikato, Christchurch, Wellington, NZ Dep Index; decile 1—least deprived to decile 10—most deprived, area of residence; rural or urban, mode of diagnosis; screened or symptomatic, stage; using AJCC 7th edition TNM staging, grade; 1—low to 3—high, histology; ductal, lobular, mixed, other, ER/PR; ER+/PR+, ER + /PR−, ER−/PR+, ER−/PR−, unknown, HER; negative, equivocal, positive, unknown, radiotherapy; radiotherapy or no radiotherapy, systemic; systemic treatment(chemotherapy, hormone therapy or biologics) or no systemic treatment

In terms of receipt of RT by treatment facility type, in private care, wāhine Māori were less likely to receive RT compared to NZE women (OR 0.62; 95% CI 0.39, 0.98), with no other notable differences for other ethnicities. Age and mode of diagnosis were key contributing factors for wāhine Māori in private care. In the public system, Pacific women were 35% less likely (OR 0.65; 95% CI 0.49, 0.85) to receive RT than NZE women. Key contributing factors to this reduction in odds differential for Pacific women were age, tumour factors and receipt of systemic therapy. (Supplementary Table S4). *χ*^2^ for heterogeneity were non-significant for this subgroup analysis.

### Sensitivity analysis

Overall, CCI was recorded for 4,084 women. 70.1% had a score of 0, 26.1% a score of 1–2, 25% a score of 3–4 and 1.4% a score ≥ 5. Inclusion of CCI in the model did not significantly alter the OR for type of surgery or receipt of RT for any ethnicity compared to NZE (Supplementary Tables S5 and S6).

## Discussion

### Key findings

This study demonstrated ethnic differences in type of surgery and receipt of RT in BCS-eligible women with early-stage breast cancer in NZ. Over one-third of women with possible BCS-eligible tumours received mastectomy. Asian women were more likely to receive mastectomy, compared to NZE women, with no significant differences for other ethnicities overall. When separated by treatment facility type, compared to NZE, wāhine Māori were more likely to receive mastectomy in the public system but not in the private system. Regarding receipt of RT, Pacific women undergoing BCS were markedly less likely to receive RT, compared to NZE women and wāhine Māori were also less likely to receive RT compared to NZE women in private care.

### Comparison to other literature

BCS + RT is the favourable surgical choice for women with early-stage breast cancer, with meta-analyses now suggesting superior cancer outcomes for BCS + RT [13, 14]. Despite this, high mastectomy rates among ethnic minority women, (who are BCS-eligible) have been demonstrated both in NZ and internationally [18, 24–26]. However, overall we found no statistically significant differences in type of surgery for wāhine Māori or Pacific women. This may suggest improvement in ethnic inequities with respect to type of surgery, given it differs from a prior study of women with breast cancer between 2005 and 2010 [18]. Of concern, are our subgroup findings, by treatment facility type for wāhine Māori, who were more likely to receive mastectomy (when BCS-eligible) in public, but not private care. This is consistent with prior studies, however in contrast with prior literature, we found no differences with respect to mode of diagnosis [18].

Tumour stage contributed to odds of mastectomy for wāhine Māori and Pacific women, as the OR for mastectomy decreased for both ethnicities when tumour stage was added to the model, which is consistent with the need to perform mastectomy with larger breast cancers that make breast conservation impractical. Similar to prior studies, Asian women were 62% more likely to have mastectomy compared to NZE. The increased mastectomy rate among Asian women also is likely to be because BCS requires an adequate breast to tumour ratio and on average, Asian women have smaller breast size [27]. In addition, the higher mastectomy rate may be contributed by on average younger age at diagnosis for Asian women, as documented previously [26].

Adjuvant RT following BCS is required to achieve local disease control and to obtain equivalent survival to mastectomy [10–12]. Our finding, that Pacific women undergoing BCS are less likely to receive RT, is consistent with recent NZ-based literature [19]. Other studies demonstrate that receipt of adjuvant RT for early stage breast cancer differs by ethnicity, levels of deprivation and age [28]. Previous studies have also shown that women from rural backgrounds who are more distant from radiation treatment facilities are less likely to undergo BCS and RT [29]. This study has shown no influence of area of residence on use of BCS and RT.

### Implications

One-third of women who are possibly BCS-eligible, still received mastectomy, which is higher than documented previously, such as a 22% and 26% in NZ-based studies between 2010–2015 and 2000–2015, respectively [18, 19]. Recent meta-analyses suggest that BCS should be seen as the favourable first-line option for these women^±^ [13,14]. Our findings may suggest that either women are not aware of this, or this has not yet been adopted in clinical practice, given the relatively high mastectomy rate. It may even suggest that the mastectomy rates are higher in recent years, given the rate is higher than in earlier studies. The absence of ethnic differences for wāhine Māori and Pacific could signify improvement with respect to inequities in type of surgery, compared to earlier studies [18]. However, given, the overall high mastectomy rate, it is unclear whether this negative finding represents an increased mastectomy rate among NZE, rather than increased BCS among wāhine Māori and Pacific women.

It is important to highlight that the oncological equivalence (or probable superiority, per recent meta-analyses of observational studies) of BCS to mastectomy is dependent on the receipt of adjuvant RT to achieve local disease control and reduce local recurrence rates [9–11, 13, 14]. It is therefore of great concern, that in our study, Pacific women undergoing BCS, were markedly less likely to receive RT not only overall, but when treated in public, but not in private care. Inversely, wāhine Māori, undergoing BCS, were less likely to receive RT in private, but not in public care. These subgroup analyses highlight an important systemic issue–treatment for early-stage breast cancer appears to differ by treatment facility type. Where differences exist in the current healthcare system, between public and private care facilities, action is needed to pull the lesser level of performance up to the higher level in either system. This is a challenge where performance is better in private, as wāhine Māori and Pacific are less likely to receive treatment for breast cancer in private care facility [30]. Māori patients also report experiences of interpersonal racism and discrimination within the NZ healthcare system, which may represent an unmeasurable explanation for these differences by treatment facility type [31, 32]. Modern guidelines strongly encourage the use of breast conserving surgery, where appropriate. We hope that this outcome will be carefully monitored both by region and ethnicity in the NZ Breast Cancer Quality Performance Indicators currently under development [33], to facilitate more standardized and equitable surgical management of early-stage breast cancer.

### Strengths and limitations

This study provides important information on ethnic inequities in type of breast cancer surgery for women with early-stage breast cancer, across four urban centres in NZ. Data from *Te Rēhita Mate Ūtaetae* facilitated a comprehensive analysis; the registry has a less than 1% withdrawal rate and is representative of 99% of eligible cancer cases [20]. It contains more detail on tumour and treatment factors compared to other national databases [21]. Use of the MOH ethnicity data protocol [22] maximized minority group representation though there was underrepresentation of wāhine Māori and Pacific women and Māori, especially in subgroup analyses.

Study limitations must be considered. The completeness of the registry for comorbidities is limited (recorded for 25%) and comorbidity may dictate type of surgery received or the appropriateness of adjuvant RT [18, 19]. However, sensitivity analysis with adjustment for CCI did not significantly change the results for either outcome. We do not have information on the location or size of the tumour relative to breast size which are important factors when considering BCS-eligibility, and instead we could only define this by considering stage, and excluding multifocal and bilateral tumours. This study did not evaluate if ethnic differences with respect to breast reconstruction exist, and future studies could consider this. Importantly, this study did not capture the reason for final choice of surgery type. For example, data on breast size were not available which dictate surgical feasibility and cosmesis outcomes, nor was total tumour size including extent of multifocal cancer or associated DCIS examined in this study. Instead, size of the largest single invasive focus was used as part of stage assignment. More importantly, we did not have information on patient choice which may introduce a differential bias as wider cultural factors may differ by ethnicity and dictate the choice of surgery. This may stem not only from different beliefs about the most effective treatment for breast cancer, but also from practical issues such as the ability to attend daily radiation treatments between 3 and 5 weeks as utilized during the period of this analysis. Future studies, such as qualitative interviews among women from ethnic minority groups would address this and would improve the representation of wāhine Māori and Pacific women, who were underrepresented in our subgroup analyses.

## Conclusion

A significant proportion of women in NZ with early-stage breast cancer underwent mastectomy, despite earlier studies demonstrating oncological equivalence of BCS + RT to mastectomy, and recently meta-analyses demonstrating superiority with respect to survival. Significant ethnic inequities and differences by treatment facility type were also demonstrated. These findings underscore the need to standardize the management of early-stage breast cancer to facilitate equity in surgical management, with a particular focus on equal provision of treatment in public and private care.

### Supplementary Information

Below is the link to the electronic supplementary material.

Supplementary file1 (DOC 136 KB)

## Data Availability

The datasets analysed during the current study are not publicly available to protect privacy and confidentiality. Data requests for de-identified data can be made online through *Te Rēhita Mate Ūtaetae* (The NZ Breast Cancer Foundation National Register).
