# Correction to: Ethnic inequities in use of breast conserving surgery and radiation therapy in Aotearoa/New Zealand: which factors contribute?

**DOI:** 10.1007/s10549-024-07402-x

**Published:** 2024-07-06

**Authors:** Leah Boyle, Ross Lawrenson, Vili Nosa, Ian Campbell, Sandar Tin Tin

**Affiliations:** 1 Cancer Epidemiology Unit, Oxford Population Health, The University of Oxford, Oxford, UK; 2 University of Waikato, Hamilton, New Zealand; 3 Waikato District Health Board, Hamilton, New Zealand; 4 Faculty of Medical and Health Sciences, University of Auckland, Auckland, New Zealand; 5 Department of Surgery, Faculty of Health Sciences, University of Auckland, Auckland, New Zealand; 6 Epidemiology and Biostatistics, School of Population Health, University of Auckland, Auckland, New Zealand

**Correction to: Breast Cancer Research and Treatment (2024) 205:641–653** 10.1007/s10549-024-07289-8

Following recent publication of this article, the authors were contacted by *Te Aho o Te Kahu – Cancer Control Agency, New Zealand* regarding concerns with reference to the agency’s breast cancer quality performance indicators, which are not yet publicly available. Additionally, the authors inaccurately stated that the agency recommend breast-conserving surgery – the agency do not provide clinical recommendations. In the new article, references to these QPIs have therefore been changed as follows.

In the abstract section, the sentence “Recently developed NZ Quality Performance Indicators strongly encourage breast conservation and should facilitate more standardized and equitable surgical management of early-stage breast cancer” is replaced with “Modern guidelines encourage BCS + RT. In NZ, this outcome must be carefully monitored by ethnicity to facilitate equitable surgical management of early-stage breast cancer.”

In the Implications section, the sentence “The NZ Quality Performance Indicators for breast cancer, strongly encourage BCS and RT where appropriate, and it is hoped the implementation and monitoring of these indicators will have a significant impact on use of BCS and RT [33].” is replaced with “Modern guidelines strongly encourage the use of breast conserving surgery, where appropriate. We hope that this outcome will be carefully monitored both by region and ethnicity in the NZ Breast Cancer Quality Performance Indicators currently under development [33], to facilitate more standardized and equitable surgical management of early-stage breast cancer.”

Further, the term *Te Rēhita Mate Ūtaetae* has been misspelt as *Te Rēhita Mata Ūtaetae* throughout the article and the same has been updated in the article.

The original article has been corrected.

