# Supplementary material for: Ethnic inequities in use of breast conserving surgery and radiation therapy in Aotearoa/New Zealand: which factors contribute?
Source: Breast Cancer Res Treat. 2024 Mar 27;205(3):641–53. doi: 10.1007/s10549-024-07289-8 (PMC11101543; doi:10.1007/s10549-024-07289-8)
Supplement: Supplementary file 1 — Supplementary file1 (DOC 136 KB) [file 10549_2024_7289_MOESM1_ESM.doc]

**Supplementary Files**

**Authors**

Leah Boyle^1^, Ross Lawrenson^2,3^, Vili Nosa^4^, Ian Campbell^5^ and Sandar Tin Tin^1,6^

**Affiliations**

1. Cancer Epidemiology Unit, Oxford Population Health, The University of Oxford, United Kingdom
2. University of Waikato, Hamilton, New Zealand.
3. Waikato District Health Board, Hamilton, New Zealand.
4. Faculty of Medical and Health Sciences, University of Auckland, New Zealand
5. Department of Surgery, Faculty of Health Sciences, University of Auckland, New Zealand
6. Epidemiology and Biostatistics, School of Population Health, University of Auckland

**Corresponding author**

Dr Leah Boyle

[leahimogenboyle@gmail.com](mailto:leahimogenboyle@gmail.com)

**Details of file**

This file contains the supplementary tables S1-S6 which are referenced in the main body.

Table S1. Categorisation of covariates

| **Domain** | **Covariates** | **Categorisation^a^** | | **Comments / Definitions** |
| --- | --- | --- | --- | --- |
|  |  | <45 years  **≥ 45 to ≤ 69 years**  >69 years | | Reference group is the screening age-group |
| Demographic | Age |  |  | Reference group is the screening age-group^b^ |
|  | Region | **Auckland**  Waikato  Christchurch  Wellington | Main urban area | Minor urban area | Secondary urban area | Inlet  | Inland Water  Rural | Rural centre  Tis N0 M0  T1 N0 M0  T0 N1 M0 | T1 N1 M0 | T2 N0 M0  T2 N1 M0 | T3 N0 M0  T0 N2 M0 | T1 N2 M0 | T2 N2 M0 | T3 N1 M0 | T3 N2 M0  T4 Any N M0  Any T N3 M0  Any T Any N M1  Low  Intermediate  High  8500/3 – Invasive carcinoma / Ductal no special type / Invasive ductal carcinoma with medullary features | 8522/4 – Pleiomorphic ductal carcinoma | 8211/3 – Tubular ductal carcinoma  8520/3 – Invasive lobular carcinoma | 8035/3 – Lobular carcinoma with osteoclast giant cells |  |
|  | NZ Deprivation Index^c,49^ | **1-2**  3-4  5-6  7-8  9-10  Unknown |  | Diagnosis date matched to census in 5-yearly intervals:  2001 for diagnoses 1/1/2000 – 31/12/2004  2006 for diagnoses 1/1/2005 – 31/12/2009  2013 for diagnoses 1/1/2010 – 31/12/2014  2018 for diagnoses 1/1/2015 – 31/12/2020 |
|  | Area of Residence | **Urban**  Rural  Unknown |  | Based on NZ Statistics Rural/Urban classification^50^  Urban includes: main urban area \| minor urban area \| secondary urban area \| inlet  Rural includes: rural \| rural centre |
|  |  |  |  |  |
| Mode of diagnosis | Mode of diagnosis | **Screened**  Symptomatic |  |  |
|  |  |  |  |  |
|  |  |  |  | American Joint Commission on Cancer 7^th^ edition – s |
| Tumour factors | TNM stage | 0  **1**  2a  2b  3a  3b  3c  4 |  | American Joint Commission on Cancer 7^th^ edition^48^ |
|  | Grade | **1**  2  3  Unknown |  |  |
|  | Morphology | **Ductal**  Lobular  Mixed  Other  Unknown |  |  |
|  | ER/PR | **ER+/PR+**  ER+/PR-  ER-/PR+  ER-/PR-  Unknown, |  |  |
|  | HER | **Negative**  Equivocal  Positive  Unknown |  | Immunohistochemical analysis (IHC) used as primary assay; Fluorescence in-situ hybridization used for IHC equivocal results |
|  |  |  |  |  |
| Treatment facility type | Treatment facility type | **Public**  Private  Unknown |  |  |
| Treatment factors | Radiotherapy | **Yes**  No | |  |
|  | Systemic therapy | **Yes**  No | Chemotherapy | Endocrine therapy | Biologics |  |

**Table S2. Multivariate logistic regression model for odds of mastectomy compared to BCS, displaying odds ratio for each covariate included in the final model**

| **Domain** | **Covariate^a^** | **OR** | | **95% CI** | |  | |
| --- | --- | --- | --- | --- | --- | --- | --- |
| Demographics  Mode of diagnosis  Tumour factors  Treatment facility type  Treatment factors | Age  <45 years  >69 years  Region  Christchurch  Waikato  Wellington  NZ Dep Index  3 – 4  5 – 6  7 – 8  9 – 10  Area of residence  Rural    Mode of diagnosis  Screened  Stage  1b  2a  2b  3a  Grade  2  3  Histology  Lobular  Mixed  Other  Receptors  ER+/PR-  ER-/PR+  ER-/PR-  HER  Equivocal  Positive    Treatment facility type  Public  Radiotherapy  No radiotherapy  Systemic therapy  No systemic therapy | 1.30  1.09  2.01  0.63  0.90  1.02  0.96  1.09  1.11  1.01  0.58  1.75  2.68  12.16  31.6  1.20  1.38  1.68  0.75  0.73  1.15  1.47  1.43  0.62  1.60  1.41  40.2  1.50 | | 1,12, 1.50  0.96, 1.24  1.73, 2.34  0.53, 0.74  0.77, 1.05  0.89, 1.17  0.84, 1.11  0.94, 1.26  0.94, 1.31  0.87, 1.19  0.52, 0.65  1.31, 2.35  2.36, 3.03  10.4, 14.3  26.3, 38.0  1.06, 1.37  1.18, 1.61  1.44, 1.96  0.57, 0.98  0.59, 0.89  0.98, 1.36  0.91, 2.37  1.23, 1.68  0.23, 1.73  1.39, 1.84  1.27, 1.57  35.8, 45.1  1.32, 1.71 | |  | |
|  |  |  |  | |  |  |  |

1. Reference groups: age: ≥45 to ≤69 years, region: Auckland, NZ Dep Index: 1 – 2, Area of residence: urban, mode of diagnosis; symptomatic, stage: 1a, grade: 1, histology: ductal, receptors: ER+/PR+, HER: negative, treatment facility type: public, radiotherapy: no radiotherapy, systemic: no systemic therapy.

Abbreviations used in the table –NZ Dep Index – New Zealand Deprivation Index, ER – Oestrogen Receptor, PR – Progesterone Receptor, HER – Human Epidermal Growth Factor Receptor, BCS – Breast Conserving Surgery, RT - radiotherapy

**Table S3. Subgroup analysis for models for receipt of radiotherapy in women receiving breast conserving surgery by for screened and symptomatic mode of diagnosis**

| **Model** | **Additional variables**  **in model^a^** | **Screened (n = 5,947)**  **OR (95% CI)** | | | | **Symptomatic (n = 4,337)**  **OR (95% CI)** | | | |  |
| --- | --- | --- | --- | --- | --- | --- | --- | --- | --- | --- |
|  |  | **NZ European**  (n=4,560) | **Māori**  (n=637) | **Asian**  (n=422) | **Pacific**  (n=328) | **NZ European**  (n= 3,321) | **Māori**  (n=413) | **Asian**  (n=375) | **Pacific**  (n=228) | |
| Unadjusted |  | 1.00 | 0.97 (0.76, 1.25) | 1.11 (0.81, 1.52) | 0.65 (0.48, 0.87) | 1.00 | 1.04 (0.78, 1.38) | 1.05 (0.77, 1.41) | 0.92 (0.64, 1.32) | |
|  |  |  |  |  |  |  |  |  |  | |
| 1. Unadjusted + Demographics | Age | 1.00 | 0.89 (0.69, 1.15) | 1.02 (0.74, 1.40) | 0.59 (0.43, 0.79) | 1.00 | 0.88 (0.65, 1.17) | 0.85 (0.62, 1.15) | 0.76 (0.52, 1.10) | |
|  | Region | 1.00 | 0.90 (0.69, 1.16) | 1.08 (0.79, 1.50) | 0.63 (0.46, 0.85) | 1.00 | 0.89 (0.66, 1.19) | 0.90 (0.66, 1.22) | 0.81 (0.56, 1.18) | |
|  | NZ Dep Index  Area of residence | 1.00  1.00 | 0.89 (0.68, 1.16)  0.89 (0.68, 1.16) | 1.09 (0.79, 1.50)  1.08 (0.78, 1.50) | 0.62 (0.45, 0.86)  0.62 (0.45, 0.86) | 1.00  1.00 | 0.86 (0.63, 1.16)  0.85 (0.63, 1.16) | 0.89 (0.65, 1.21)  0.90 (0.66, 1.23) | 0.79 (0.54, 1.16)  0.79 (0.54, 1.17) | |
|  |  |  |  |  |  |  |  |  |  | |
| 2. Model 1 + Tumour factors | Stage | 1.00 | 0.87 (0.66 1.13) | 1.08 (0.78, 1.49) | 0.60 (0.43, 0.82) | 1.00 | 0.84 (0.62, 1.14) | 0.90 (0.66, 1.23) | 0.76 (0.52, 1.12) | |
|  | Grade | 1.00 | 0.88 (0.67, 1.16) | 1.07 (0.77, 1.48) | 0.58 (0.42, 0.80) | 1.00 | 0.84 (0.62, 1.14) | 0.90 (0.66, 1.23) | 0.76 (0.51, 1.12) | |
|  | Histology | 1.00 | 0.87 (0.66, 1.14) | 1.04 (0.75, 1.44) | 0.57 (0.41, 0.78) | 1.00 | 0.85 (0.63, 1.16) | 0.90 (0.66, 1.24) | 0.76 (0.52, 1.13) | |
|  | ER/PR | 1.00 | 0.88 (0.67, 1.15) | 1.06 (0.76, 1.47) | 0.57 (0.41, 0.78) | 1.00 | 0.85 (0.63, 1.16) | 0.91 (0.67, 1.25) | 0.78 (0.53, 1.15) | |
|  | HER | 1.00 | 0.88 (0.68, 1.16) | 1.06 (0.77, 1.48) | 0.57 (0.41, 0.79) | 1.00 | 0.85 (0.63, 1.16) | 0.90 (0.66, 1.24) | 0.78 (0.53, 1.15) | |
| 3. Model 2 + Treatment facility | Treatment facility | 1.00 | 0.84 (0.64, 1.10) | 1.04 (0.75, 1.44) | 0.53 (0.38, 0.74) | 1.00 | 0.88 (0.65, 1.20) | 0.94 (0.68, 1.29) | 0.83 (0.56, 1.22) | |
| 4. Model 3 + Treatment factors | Systemic | 1.00 | 0.82 (0.62, 1.08) | 1.01 (0.72, 1.40) | 0.52 (0.38, 0.73) | 1.00 | 0.89 (0.65, 1.22) | 0.88 (0.64, 1.21) | 0.83 (0.56, 1.24) | |

1. Variables are categorized as follows: age; <45 years, ≥45 to ≤ 69 years (women eligible for BSA) and >69 years, region; Auckland, Waikato, Christchurch, Wellington, NZ Dep Index; decile 1- least deprived to decile 10 – most deprived, area of residence; rural or urban, mode of diagnosis; screened or symptomatic, stage; using AJCC 7^th^ edition TNM staging, grade; 1 – low to 3 – high, histology; ductal, lobular, mixed, other, ER/PR; ER+/PR+, ER+/PR-, ER-/PR+, ER-/PR-, unknown, HER; negative, equivocal, positive, unknown, systemic; systemic treatment(chemotherapy, hormone therapy or biologics) or no systemic treatment.

Abbreviations used in the table – OR – Odds Ratio, CI – Confidence Interval, NZ Dep Index – New Zealand Deprivation Index, ER – Oestrogen Receptor, PR – Progesterone Receptor, HER – Human Epidermal Growth Factor Receptor

**Table S4. Subgroup analysis for models for receipt of radiotherapy in women receiving breast conserving surgery by ethnicity in public and private treatment facilities**

| **Model** | **Additional variables**  **in model^a^** | **Public care (n = 6,176)**  **OR (95% CI)** | | | | **Private care (n = 3,952)**  **OR (95% CI)** | | | |  |
| --- | --- | --- | --- | --- | --- | --- | --- | --- | --- | --- |
|  |  | **NZ European**  (n=4,277) | **Māori**  (n=890) | **Asian**  (n=507) | **Pacific**  (n=502) | **NZ European**  (n= 3,473) | **Māori**  (n=156) | **Asian**  (n=271) | **Pacific**  (n=52) | |
| Unadjusted |  | 1.00 | 1.09 (0.88, 1.35) | 1.24 (0.93, 1.65) | 0.79 (0.61, 1.01) | 1.00 | 0.78 (0.50, 1.21) | 0.91 (0.64, 1.30) | 0.71 (0.34, 1.47) | |
|  |  |  |  |  |  |  |  |  |  | |
| 1. Unadjusted + Demographics | Age | 1.00 | 0.93 (0.75, 1.16) | 1.05 (0.79, 1.41) | 0.66 (0.51, 0.85) | 1.00 | 0.69 (0.45, 1.08) | 0.82 (0.57, 1.18) | 0.62 (0.30, 1.28) | |
|  | Region | 1.00 | 0.96 (0.77, 1.19) | 1.10 (0.82, 1.47) | 0.69 (0.53, 0.90) | 1.00 | 0.67 (0.42, 1.03) | 0.88 (0.61, 1.27) | 0.66 (0.32, 1.37) | |
|  | NZ Dep Index  Area of residence | 1.00  1.00 | 0.96 (0.77, 1.21)  0.96 (0.76, 1.21) | 1.09 (0.81, 1.47)  1.10 (0.81, 1.48) | 0.70 (0.53, 0.92)  0.70 (0.54, 0.92) | 1.00  1.00 | 0.65 (0.42, 1.02)  0.66 (0.42, 1.02) | 0.87 (0.61, 1.26)  0.87 (0.60, 1.25) | 0.62 (0.30, 1.30)  0.62 (0.30, 1.30) | |
|  |  |  |  |  |  |  |  |  |  | |
| 2. Model 1 + Mode of diagnosis | Mode of diagnosis | 1.00 | 0.96 (0.77, 1.21) | 1.12 (0.83, 1.51) | 0.71 (0.54, 0.93) | 1.00 | 0.65 (0.42, 1.02) | 0.87 (0.60, 1.24) | 0.62 (0.30, 1.29) | |
| 3. Model 2 + Tumour factors | Stage | 1.00 | 0.95 (0.75, 1.19) | 1.12 (0.83, 1.51) | 0.68 (0.52, 0.89) | 1.00 | 0.65 (0.42, 1.03) | 0.87 (0.60, 1.25) | 0.60 (0.29, 1.27) | |
|  | Grade | 1.00 | 0.95 (0.76, 1.20) | 1.12 (0.83, 1.51) | 0.67 (0.51, 0.88) | 1.00 | 0.64 (0.41, 1.00) | 0.85 (0.59, 1.23) | 0.56 (0.27, 1.19) | |
|  | Histology | 1.00 | 0.96 (0.76, 1.20) | 1.12 (0.83, 1.51) | 0.67 (0.51, 0.88) | 1.00 | 0.63 (0.40, 0.99) | 0.83 (0.58, 1.21) | 0.56 (0.27, 1.18) | |
|  | ER/PR | 1.00 | 0.96 (0.76, 1.21) | 1.13 (0.84, 1.52) | 0.67 (0.51, 0.88) | 1.00 | 0.61 (0.39, 0.96) | 0.85 (0.59, 1.23) | 0.56 (0.27, 1.19) | |
|  | HER | 1.00 | 0.95 (0.75, 1.19) | 1.11 (0.83, 1.50) | 0.67 (0.51, 0.88) | 1.00 | 0.62 (0.39, 0.98) | 0.87 (0.60, 1.26) | 0.57 (0.27, 1.20) | |
| 5. Model 3 + Treatment factors | Systemic | 1.00 | 0.93 (0.74, 1.17) | 1.05 (0.78, 1.43) | 0.65 (0.49, 0.85) | 1.00 | 0.62 (0.39, 0.98) | 0.86 (0.59, 1.24) | - 1. (0.28, 1.27) | |

1. Variables are categorized as follows: age; <45 years, ≥45 to ≤ 69 years (women eligible for BSA) and >69 years, region; Auckland, Waikato, Christchurch, Wellington, NZ Dep Index; decile 1- least deprived to decile 10 – most deprived, area of residence; rural or urban, mode of diagnosis; screened or symptomatic, stage; using AJCC 7^th^ edition TNM staging, grade; 1 – low to 3 – high, histology; ductal, lobular, mixed, other, ER/PR; ER+/PR+, ER+/PR-, ER-/PR+, ER-/PR-, unknown, HER; negative, equivocal, positive, unknown, systemic; systemic treatment(chemotherapy, hormone therapy or biologics) or no systemic treatment.

Abbreviations used in the table – OR – Odds Ratio, CI – Confidence Interval, NZ Dep Index – New Zealand Deprivation Index, ER – Oestrogen Receptor, PR – Progesterone Receptor, HER – Human Epidermal Growth Factor Receptor

**Table S5. Sensitivity analysis – logistic regression models for type of surgery, by ethnicity including adjustment for comorbidities**

| **Model** | **NZ European**  (n=2,983) | **Māori**  (n=485) | **Asian**  (n=376) | **Pacific**  (n=240) |
| --- | --- | --- | --- | --- |
|  | **Reference** | **OR (95% CI)** | | |
| Maximally adjusted^a^  Maximally adjusted + comorbidity score^b^ | 1.00  1.00 | 1.37 (1.02, 1.84)  1.34 (1.00, 1.80) | 1.66 (1.20, 2.29)  1.67 (1.21, 2.31) | 1.06 (0.70, 1.60)  1.03 (0.68, 1.56) |

1. Maximally adjusted model includes adjustment for demographic factors (age, region, NZ Deprivation Index, area of residence), mode of diagnosis, tumour factors (stage, grade, morphology, ER/PR - oestrogen/progesterone receptors, HER - Human Epidermal Growth Factor Receptor), treatment facility type and treatment factors (radiotherapy, systemic therapy)
2. Comorbidity score using Charlson Comorbidity Index categorized as: 0, 1-2, 3-4 and ≥5 [23]

**Table S6. Sensitivity analysis – logistic regression models for receipt of RT, by ethnicity including adjustment for comorbidities**

| **Model** | **NZ European**  (n=2,983) | **Māori**  (n=485) | **Asian**  (n=376) | **Pacific**  (n=240) |
| --- | --- | --- | --- | --- |
|  | **Reference** | **OR (95% CI)** | | |
| Maximally adjusted^a^  Maximally adjusted + comorbidity score^b^ | 1.00  1.00 | 0.94 (0.66, 1.34)  0.92 (0.65, 1.31) | 1.25 (0.80, 1.96)  1.25 (0.80, 2.00) | 1.09 (0.64, 1.86)  1.08 (0.64, 1.84) |

1. Maximally adjusted model includes adjustment for demographic factors (age, region, NZ Deprivation Index, area of residence), mode of diagnosis, tumour factors (stage, grade, morphology, ER/PR - oestrogen/progesterone receptors, HER - Human Epidermal Growth Factor Receptor), treatment facility type and treatment factors (systemic therapy)
2. Comorbidity score using Charlson Comorbidity Index categorized as: 0, 1-2, 3-4 and ≥5 [23]
